# New Health Care Reform and Impoverishment among Chronic Households in China: A Random-Intercept Approach

**DOI:** 10.3390/ijerph16061074

**Published:** 2019-03-26

**Authors:** Yongjian Xu, Anupam Garrib, Zhongliang Zhou, Duolao Wang, Jianmin Gao, Xiaowei Yang, Xiaojing Fan, Gang Chen

**Affiliations:** 1 School of Public Policy and Administration, Xi’an Jiaotong University, Xi’an 710049, China; wgsxyj@xjtu.edu.cn (Y.X.); zzliang1981@xjtu.edu.cn (Z.Z.); gaojm@mail.xjtu.edu.cn (J.G.); yangxwde@mail.xjtu.edu.cn (X.Y.); 2 Department of Clinical Sciences, Liverpool School of Tropical Medicine, Liverpool L3 5QA, UK; Anupam.Garrib@lstmed.ac.uk (A.G.); fanxj112@xjtu.edu.cn (X.F.); 3 School of Public Health, Health Science Center, Xi’an Jiaotong University, Xi’an 710049, China; 4 Centre for Health Economics, Monash Business School, Monash University, Melbourne, VIC 3800, Australia; gang.chen@monash.edu

**Keywords:** China, new health care reform, impoverishment, chronic diseases, out-of-pocket expenditure

## Abstract

High out-of-pocket (OOP) payments for chronic disease care often contribute directly to household poverty. Although previous studies have explored the determinants of impoverishment in China, few published studies have compared levels of impoverishment before and after the New Health Care Reform (NHCR) in households with members with chronic diseases (hereafter referred to as chronic households). Our study explored this using data from the fourth and fifth National Health Service Surveys conducted in Shaanxi Province. In total, 1938 households in 2008 and 7700 households in 2013 were included in the analysis. Rates of impoverishment were measured using a method proposed by the World Health Organization. Multilevel logistic modeling was used to explore the influence of the NHCR on household impoverishment. Our study found that the influence of NHCR on impoverishment varied by residential location. After the reform, in rural areas, there was a significant decline in impoverishment, although the impoverishment rate remained high. There was little change in urban areas. In addition, impoverishment in the poorest households did not decline after the NHCR. Our findings are important for policy makers in particular for evaluating reform effectiveness, informing directions for health policy improvement, and highlighting achievements in the efforts to alleviate the economic burden of households that have members with chronic diseases.

## 1. Introduction

Chronic noncommunicable diseases (NCDs) are a major global public health concern and accounted for over 70% of the world’s 56.9 million deaths in 2016 [1,2]. Cardiovascular diseases, cancers, diabetes, and chronic lung diseases are four major NCDs that have imposed the heaviest disease burden. The burden of NCDs is increasing disproportionately in low- and middle-income countries where, according to World Health Organization (WHO) estimates, more than three quarters of NCD deaths occurred in 2016 [2]. China has experienced rapid socioeconomic and environmental changes in recent decades. Rapid urbanization, changes in diet, and high levels of tobacco use, along with an aging population, have contributed to an alarming rise in the prevalence of NCDs. China’s National Health and Family Planning Commission (NHFPC) estimated that the proportion of adults with chronic diseases increased from 12.33% in 2003 to 24.52% in 2013 and is expected to increase further [3]. NCDs are the leading cause of death and disability in China, with nearly 90% of total deaths attributed to chronic diseases [4].

Chronic diseases directly and indirectly impose an enormous economic burden on Chinese society. Households that have any member with a chronic disease (hereafter to as chronic households), including hypertension, diabetes, or cancers, are often affected by both out-of-pocket (OOP) expenditures for health services and income loss from an inability to work [5]. High OOP health expenditures force chronic households to limit their spending on other essentials, potentially leading households into poverty. Chinese government data show that health-payment-induced poverty accounted for 42.2% of total poverty in 2013 [6].

Despite significant efforts by the Chinese government to reduce the burden on chronic households before 2009, Chinese citizens still faced difficulties regarding access to and cost of health care [7]. In 2009, the Chinese government launched a new round of health system reforms, called the New Health Care Reform (NHCR). The overall goal of the reform is to establish and improve the basic health care system for both urban and rural residents and to provide residents with secure, efficient, convenient, and affordable health care services. The plan is a comprehensive reform anchored in four interdependent areas: public health, health care services, medical security or public health insurance, and secure pharmaceutical supplies. Investment in the health system has been enhanced to achieve the reform goals. From 2007 to 2014, the share of government health spending on total health spending increased from 20.3% to 29.90% [8]. Among all of the measures employed in the four areas covered by the NHCR, three major measures were most likely to address the problem of costs to access health services.

Firstly, after the NHCR, China rapidly scaled up coverage of basic health insurance schemes, covering 95% of the population by 2011 [9]. Three types of basic health insurance programs were designed for urban and rural residents to help avoid financial catastrophe caused by unexpected or expensive medical events [10]. The Urban Employee Basic Medical Insurance (UEBMI) and the Urban Resident Basic Medical Insurance (URBMI) were designed for urban employees and unemployed residents, respectively. The New Rural Cooperative Medical Insurance (NRCMI), set up in 2003, was designed to provide financial risk protection for illness in rural residents. As coverage improved, some insurance schemes also expanded the range of services for vulnerable patients by establishing supplement reimbursement policies based on existing insurance schemes. For example, a benefit package for chronic disease outpatients was added to the NRCMI in many provinces after the introduction of the NHCR.

Secondly, a national essential medicine system was established to reduce the cost of medicines and promote access to safe and effective essential drugs. A National Essential Medicines List for commonly occurring conditions has been published by the central and provincial governments. All government-run primary health institutions should be equipped with listed essential medicines only, all provided at zero markup [11].

Thirdly, a package of free medical treatments and public health interventions has been provided to reduce the economic burden on vulnerable groups. Included are interventions aimed at reducing the increasing burden of chronic NCDs, for example, free annual health check-ups for those over 65 years old, free establishment of electronic health records for residents, free cataract operations for poor patients, and regular hypertension and diabetes follow-up visits.

Numerous studies have explored the relationship between chronic diseases and health expenditure in mainland China [12,13,14]. While many of these studies investigated the economic burden of different chronic diseases on individuals, only a few studies focused on the adverse financial consequences caused by health payments in households with members having chronic conditions [15,16]. To explore health-payment-induced impoverishment, most previous studies have focused on two aspects of household impoverishment with the use of one wave of cross-sectional data in mainland China. Some explored the determinants of household impoverishment based on classical regression models [17,18]. Others explored the extent to which impoverishment would decrease after reimbursement from health insurance schemes in China [16]. So far, no published studies have investigated the influence of the NHCR on household impoverishment in China. Our study has two objectives: firstly, to investigate the occurrence of impoverishment in both urban and rural areas before and after NHCR; secondly, to explore the possible influence of NHCR on impoverishment controlling for other determinants of impoverishment. Our study contributes to the existing studies by helping us understand the heterogeneity of the effects on impoverishment and providing policy makers with evidence to evaluate policy impacts and inform policy development. 

Our study found that, although the impoverishment rate in rural areas of Shaanxi Province dropped from 13.17% to 9.11% after the implementation of the NHCR, the impoverishment rate was still considerably high in rural areas after reform. Furthermore, impoverishment in urban areas was nearly unchanged. The influence of the NHCR on impoverishment were dramatically different in urban and rural areas.

## 2. Materials and Methods

### 2.1. Study Design

This study used a repeated cross-sectional design to determine the prevalence of impoverishment in both urban and rural areas before and after the introduction of a policy reform measure (the NHCR). 

### 2.2. Data

This study used data from China’s fourth and fifth National Health Service Surveys (NHSS) conducted in Shaanxi Province. The NHSS is a large-scale, multiround, five-year survey conducted on a representative sample of households throughout China. The fourth and fifth NHSS were conducted in June 2008 and September 2013, respectively. A four-stage, stratified, random sampling method was used in both 2008 and 2013. In the survey, a face-to-face interview was conducted by interviewers using a structured household questionnaire that was developed by the National Health and Family Planning Commission. Details concerning the questionnaire contents of the NHSS and quality assurance measures were elaborated in previous studies [19,20]. Ethics approval for this study was obtained from Health Science Center of Xi’an Jiaotong University (No. 2015-644). Informed consent for the survey was obtained from the household head initially and then from other household members. In total, 5960 households in 2008 and 20,700 households in 2013 were identified in Shaanxi Province. An increased investment in the National Health Service Survey in Shaanxi Province in 2013 enabled the much larger sample size seen in that year compared with 2008. The sampled households in both years were representative of the demographic and socioeconomic characteristics of the whole population in Shaanxi Province. The structure of the population sampled in 2008 and 2013 was very similar to that derived from the population census in 2010. Further triangulation with data from the routine health information system found that estimates of health facility use at, for example, the township level, were comparable with estimations of use at the same level from routine data. The tested Myer’s Blended index was 1.67 in 2008 and 1.62 in 2013, indicating that there was no significant age preference in the survey. These findings indicate the representativeness of the sample and reliability of the data. 

This study only focused on chronic households, defined as those with at least one member suffering from a chronic noncommunicable disease. Exclusion criteria were: (1) households with missing data on health consumption and overall consumption expenditure; (2) households with missing data on household scale; (3) households with net expenditures less than $48.39 USD (RMB 300 Yuan, all U.S. dollar equivalents in the study were calculated based on the World Bank (WB) annual exchange rate in 2013 of US$ 1 = RMB 6.20 Yuan). After imposing exclusion criteria, 1938 chronic households in 2008 and 7700 chronic households in 2013 were used in this analysis.

### 2.3. Outcome Variables

#### 2.3.1. Household Impoverishment

Although policy researchers have described impoverishment that occurs when households become trapped in poverty after OOP health payments, there are no explicit methods to calculate this type of impoverishment. The two most frequently used methods to calculate impoverishment are from the WHO and WB. The WHO method uses a relative poverty line to calculate impoverishment, comparing the living standards of an individual with the rest of society, which therefore changes as the standard of living shifts. The WB method uses an absolute poverty line of $1.25 that remains consistent over time to calculate impoverishment. This absolute poverty line remains the same regardless of changes in the overall standard of living or income distribution in a society. China’s economy has been growing at almost 10% since it embraced economic reforms and free-market principles. As our study aims to compare the relative poverty before and after the NHCR, we used the WHO’s proposed method [21]. A nonpoor household is impoverished by OOP health expenditures when it becomes poor after paying for health services. For example, if the minimum expenditure required to maintain basic life in society for a person (poverty line) is $600, a household with five members that has a total expenditure of $5000 and OOP health expenditures of $2000 in the last year would be considered impoverished because the remaining household expenditures were lower than basic subsistence expenditures for this household of $3000 ($600 × 5 persons).

A variable, impoverishment, was established to reflect the effect of OOP health payments and was defined as 1 when household expenditures were equal to or higher than subsistence expenditures but lower than subsistence expenditures net of OOP health payments, and 0 otherwise:

(1) $$impoor_{h}=\{\begin{array}{l} 1\;exp_{h}\geq se_{h}\;|\&exp_{h}-oop_{h}<se_{h}|\\ 0\;otherwise \end{array}$$

where $impoor_{h}$ stands for household impoverishment, $exp_{h}$ is household total expenditures, $oop_{h}$ is household OOP health expenditures, and $se_{h}$ is the household subsistence expenditures for each household. The household subsistence expenditures are the minimum requirement to maintain basic life in a society. The household subsistence expenditures could be obtained from the following formula:

(2) $$se_{h}=pl\times eqsize_{h}$$

where pl is subsistence expenditures per (equivalent) capita, which is also the poverty line. The poverty line can be obtained by weighted average food expenditures of the household in the 45–55 percentile range:

(3) $$pl=\frac{\sum^{\;}w\times eqfood}{\sum^{\;}w}\;food45<foodexp_{h}<food55$$

where eqfood is the equalized food expenditures, which can be obtained by dividing household food expenditures by the equivalent household size. The equivalent household size can be obtained from the following formula:

(4) $$eqsize=hhsize^{0.56}$$

where eqsize is the equivalent household size, and hhsize is the actual scale of the household.

#### 2.3.2. Catastrophic Health Expenditure (CHE)

OOP health expenditures are taken to be catastrophic when a household is required to cut its basic expenditures over a period of time to cope with health payments. The WHO defines catastrophic heath expenditures as those which occur when a household’s total OOP health payments equal or exceed 40% of the household’s capacity to pay (ctp) or nonsubsistence expenditures [21]:

(5) $$ctp_{h}=\{\begin{array}{c} exp_{h}-se_{h}\;\;\;\;\;\;\;\;\;se_{h}\leq food_{h}\\ exp_{h}-food_{h}\;\;\;se_{h}>food_{h} \end{array}$$

where $ctp_{h}$ is the household’s capacity to pay, and $food_{h}$ is the household food expenditures.

### 2.4. Variables of Interest

Time is a binary variable representing the time before and after the NHCR. Residential location is a dummy variable that takes the value of 1 if the household was located in a rural area and 0 if located in an urban area. 

### 2.5. Confounding Variables

Three types of confounding variables were identified in the study. Firstly, household characteristics included three variables: household economic status (poorest, poorer, middle, richer, richest), household size (1, 2–4, ≥5), having elderly members, and having children. Household economic status was measured by per capita net household expenditures, which was used as a proxy for income. It was calculated as total household expenditures minus health expenditures divided by the number of household members. All households were ranked according to per capita net household expenditures and equally divided into five groups [22]. Having elderly members was a binary variable indicating whether there are elderly members aged 60 years old or over in the household. Having children was a binary variable indicating whether there were children aged 5 years old or under in the household. Secondly, since the head of household plays a prominent role in the household, five variables related to characteristics of the household head were also included in our analysis: age (<45, 45–60, ≥60), gender, education attainment (illiteracy, elementary, middle school, or high school and above), marital status (married, unmarried), and working status. Thirdly, types of chronic conditions included two variables: hypertension and diabetes. They were both binary variables representing whether there were any household members who had doctor-diagnosed hypertension or diabetes, respectively.

### 2.6. Statistical Analysis

The data used were collected using a multistage stratified sampling method. Such data may have hierarchical or clustered structures. Conventional statistical methods may ignore the correlation of health outcomes within clusters and are prone to underestimate standard errors in dealing with such data. However, multilevel models can partition the total variance into corresponding variance components of a hierarchical structure and subsequently obtain more accurate and unbiased estimates and standard errors. In this study, a multilevel logistic regression model was used to explore the influence of the NHCR on household impoverishment. A null model without any covariates but only an intercept and random effects at the group level was firstly established to check if there was some multilevel structure in the data. From the null model results, we found there was statistical significance in variances at the county and town levels. In addition, the variance partition coefficient was 0.3129, meaning that 31.29% of residual variance in the propensity to be impoverished was attributed to unobserved area-level characteristics. Therefore, a three-level random intercept logistic regression model was applied to estimate the influence of the NHCR on impoverishment:

(6) $$\mathrm{logit}\left(p_{ijk}\right)=\beta_{0}+\beta_{1}time_{ijk}+\beta_{2}loc_{ijk}+\beta_{3}time\times loc_{ijk}+X_{ijl}\gamma +Z_{ijk}\delta +\mu_{0jk}+\upsilon_{0k}+\varepsilon_{ijk}$$

where *i, j*, and *k* indices, respectively, represent levels 1, 2, and 3; $p_{ijk}$ is the probability of incurring impoverishment; time is a dummy variable representing the time before and after the NHCR; loc is a dummy variable that takes the value of 1 if the household is located in a rural area and 0 if located in an urban area; time×loc is an interaction term between time and residential location in the regression model to test whether the effect of the NHCR on household impoverishment varies between urban and rural areas; *γ* is the vector of household characteristics, such as household scale or having elderly members; *δ* is a vector of characteristics of the household head; $\upsilon_{0k}\;$ and $\mu_{0jk}$ are random effects at the county level (level 3) and town level (level 2), respectively.

Odds ratios (ORs) with corresponding 95% confidence intervals (CIs) were calculated in the model. The bootstrap method was employed to estimate the standard errors. All statistical analyses were conducted using Stata 12.0 (Stata Corporation, College Station, TX, USA). Two-tailed tests with a significance level of 5% were employed in statistical significance testing.

## 3. Results

### 3.1. Descriptive Statistics

Table 1 presents characteristics of chronic households and their household heads. The mean household size reduced between 2008 and 2013 in both urban and rural areas, from 2.89 to 2.85 in urban and 3.17 to 2.90 in rural areas. The mean household head age increased between 2008 and 2013 in both urban and rural areas, from 57.62 to 58.56 years old in urban and 52.29 to 55.62 years old in rural areas. The per capita net household expenditure in urban areas increased from $1097 in 2008 to $2038 in 2013, while in rural areas these figures were from $596 in 2008 to $1414 in 2013. The proportion of female household heads between 2008 and 2013 decreased from 37.43% to 31.83% in urban areas, however, these figures increased from 18.95% to 21.03% in rural areas. Although income increases were seen in urban and rural areas, the proportion of poorest households in urban areas increased while it decreased in rural areas. A possible reason for this is that with the free flow of residents from rural to urban areas, the urban/rural income ratio fell between 2008 and 2013. 

Table 2 shows the average household OOP health expenditures, average household total expenditures, the number and proportion of households with CHEs, and the household impoverishment rate in paying for health care among different economic statuses in urban and rural areas. The share of OOP health expenditures of total expenditures in both years varied across economic groups. The poorest groups had the highest percentage of OOP health expenditures, the highest proportion of households with CHEs, and the highest impoverishment rate in both urban and rural areas. The poorer group had the largest drop in impoverishment rates among all economic groups from 2008 to 2013 (*χ^2^* = 179.32, *p* < 0.001), although there was still a high proportion of households that became poor after paying for OOP health expenditures among the poorest groups from 2008 to 2013 (*χ^2^* = 4.79, *p* = 0.029).

**Figure 1:**
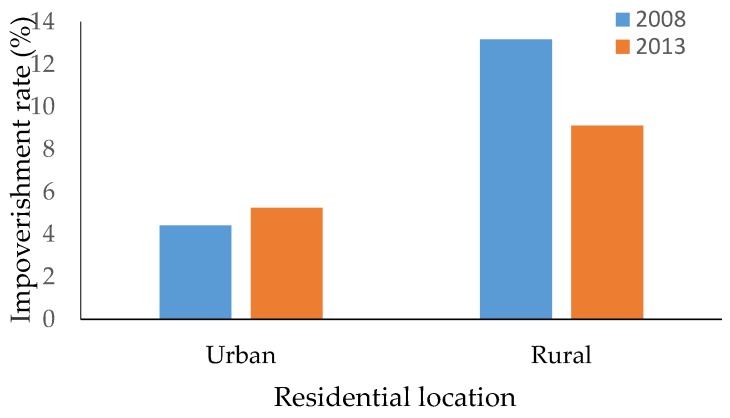
Impoverishment rate for urban and rural households in 2008 and 2013.

Figure 1 shows the impoverishment rates in urban and rural areas before and after the NHCR. Household impoverishment in urban areas was consistently lower than in rural areas in both 2008 and 2013. For urban households, the impoverishment rate was 4.42% and 5.26% in 2008 and 2013, respectively, although this increase was not statistically significant (*χ^2^* = 1.0718, *p* = 0.301). For households in rural areas, the impoverishment rate decreased from 13.17% in 2008 to 9.11% in 2013, a significant change in impoverishment rates (*χ^2^* = 15.329, *p* < 0.001).

### 3.2. Results of the Model

Table 3 presents the estimated odds ratios of the three-level logit model. After controlling for household and household head characteristics, the random intercept model showed that the main effect of time was not significant, while the interaction between time and residential location of residence was significant. This implies that the effects of the NHCR on the occurrence of impoverishment were heterogeneous between rural and urban households. The heterogeneous effects of the NHCR on impoverishment by urban and rural residence are displayed in Table 4. The analysis showed that there was a significant before-and-after difference in the odds of impoverishment in rural areas (OR: 0.54, 95% CI: 0.43–0.68, *p* < 0.001), but there was no difference in urban areas (OR: 0.83, 95% CI: 0.56–1.23, *p* = 0.35).

## 4. Discussion

Unlike our published study which compared catastrophic health expenditures before and after the NHCR, the main objective of this study was to evaluate whether the NHCR helped to alleviate impoverishment in rural and urban households [23]. After controlling for covariates in the model, our study found that the influence of the NHCR on impoverishment varied by residential location. The impoverishment rate in rural households dropped significantly from 2008 to 2013, while the impoverishment in urban areas changed little over the same period. It is likely that several factors have contributed to this phenomenon. Firstly, the basic medical insurance benefits grew faster in rural areas than in urban areas. Although urban medical insurance schemes are generous, the reimbursement ratio dramatically increased in rural areas in recent years with NRCMI policy optimization [24]. A special reimbursement policy for specific chronic conditions in the NRCMI was added to the NHCR, which had a significant effect on the economic burden of health care for chronic disease households in rural areas [25]. In addition, several prospective payment methods under the NRCMI were piloted to control medical costs in some rural counties of Shaanxi Province, such as Mei county. Fee-for-service was the predominant insurance payment method in Shaanxi Province and has long driven up health care costs. Implementation of prospective payments, such as global budgets and per diem reimbursement, has decreased the growth rate of health care spending in piloted areas. Secondly, a zero-markup policy for medicines was introduced in primary health care institutions after the reform to decrease the costs of medicines. Since a higher proportion of rural residents were able to access health services in primary health care institutions than urban residents, rural residents benefited more from this policy. 

Previous studies concerning impoverishment have focused predominantly on the effect of health insurance on reducing impoverishment rates through comparing the health-payment-induced poverty before and after reimbursement using one set of cross-sectional survey data [16,20,26]. Few studies have compared the impoverishment rate before and after a wider health policy reform such as the NHCR, which includes several elements in addition to health insurance. Before and after reimbursement studies have some limitations. Since the insured received their reimbursement from health insurance, their household impoverishment rate would almost certainly decline. As the effect of different policies on impoverishment may overlap, cancel each other out, or otherwise interact, our study provides evidence on whether the NHCR was helpful in reducing impoverishment in chronic households using repeated cross-sectional data.

Our study found that lower economic status households had a higher likelihood of experiencing impoverishment, a finding consistent with other studies [27]. That is because lower-income households lack the ability to protect against catastrophic health expenditures caused by chronic diseases and their complications. The study also found that chronic households in rural areas had a higher proportion of impoverishment than in urban areas in both 2008 and 2013. Our unpublished data showed that urban residents used more health services than rural residents; the hospitalization rate was 10.47% in urban and 9.82% in rural areas, and the two-week hospital visit rate was 13.56% in urban and 11.96% in rural areas in 2013. The higher use of health services and lower incidence of impoverishment by urban households could be explained by the higher income of urban residents. Statistics have shown that there is a big income gap between urban and rural areas in China [28]. The annual per capita disposable income of urban households was nearly three times more than that of rural households in 2015, at $5031 and $1842, respectively [27]. Therefore, urban households had a greater capacity to withstand the financial impact of ill-health. 

Our study also found that, although the overall impoverishment rate declined after the NHCR, impoverishment in the poorest households increased in both urban and rural areas. There was also little change in CHEs before and after the NHCR among different economic groups, and the poorest households had the highest incidence of CHEs in both years. That means the poorest benefited the least from this reform compared with other economic groups. A possible reason for this is that, although many financial risk protection measures were implemented in Shaanxi Province, the cost of health services also increased; therefore, accessing health services still imposed a heavy financial burden. Further attention needs to be given to ensuring that the poorest are able to benefit more from these reforms. 

Our findings have some important policy implications for reducing impoverishment in developing countries [29,30]. The NCHR included four main policy elements, all of which could potentially contribute to reducing impoverishment in households where a member has a chronic illness. Firstly, basic health insurance protects a household’s members against the financial burden of paying for health care, particularly if care will be ongoing, as in the case of chronic diseases. Where basic health insurance provides a low level of protection for residents, establishing supplementary reimbursement policies for specific populations, such as those with chronic conditions, helps provide additional support to those vulnerable groups. Replacing traditional fee-for-service payment with prospective payments could also protect residents from the potentially devastating effects of health care costs. Secondly, developing a two-way referral system between hospitals and primary health care systems can also reduce OOP health expenditures. Before the NHCR, people were free to access health services at any health facility of their choice. This meant that many sought health care at more costly tertiary institutions rather than at lower-cost primary health care institutions, regardless of their condition. With the NHCR, a hierarchical medical system has been established that allows two-way referrals between hospitals and primary care facilities, although referrals tend to be from primary care facilities to hospitals; referrals from hospitals to the community is rare in China [31]. Therefore, development of two-way referral systems could not only alleviate the difficulty in accessing health services but also decrease the health economic burden for urban and rural residents. Thirdly, costs of accessing medication can be a significant problem for those with chronic diseases, as it represents an ongoing expenditure. Having essential medication available a low or no cost can represent a significant cost saving for persons with chronic conditions. 

Future policy developments should be targeted to the poorest households which have the highest risk of suffering from impoverishment and seem to have benefited the least from the protections afforded by the NHCR. Further study of the reasons for this is needed to inform appropriate strategies to enable the poorest households to benefit more. 

There are some limitations to the present study. Firstly, as repeated cross-sectional data were used to explore the influence of the NHCR on impoverishment, no causal inference can be drawn from this analysis. Secondly, the NHCR was simultaneously implemented across mainland China; therefore, current control groups could not be used in the study. The observed differences may, therefore, be subject to possible unobserved confounding factors. Thirdly, in the fourth and fifth NHSS, all the information was self-reported, resulting in possible recall bias in the data. Fourthly, Although the NHHS collected information on overall outpatient and inpatient health service utilization, more detailed information on what kind of services the respondents used were not reported, so we are unable to comment on the types of services used that may have led to impoverishment.

## 5. Conclusions

In conclusion, the NHCR may be helpful to protect chronic households from potential impoverishment due to payments for health care. However, the effects of the NHCR on the occurrence of impoverishment were heterogeneous between rural and urban areas. While the impoverishment rate in rural households had a significant decline after the reform, the impoverishment rate in urban areas showed little change. Our study suggests that the elements of the NHCR that include establishing a special reimbursement policy for chronic disease members under the existing public health insurance scheme, subsidizing drug costs, and developing referral systems between high-level health institutions and primary health institutions could be helpful in protecting urban and rural residents from the potentially devastating effects of health care spending.

## Figures and Tables

**Table 1:** Characteristics of chronic households and household heads (mean ± SD or number (%)).

Variables	*n*	Urban		Rural	
		Before Reform (*n* = 951)	After Reform (*n* = 2868)	Before Reform (*n* = 987)	After Reform (*n* = 4832)
Household scale					
	2.91 ± 1.36	2.89 ± 1.23	2.85 ± 1.31	3.17 ± 1.46	2.90 ± 1.38
1	1031	100 (10.52)	294 (10.25)	97 (9.83)	540 (11.18)
2–4	7208	729 (76.66)	2189 (76.32)	705 (71.43)	3585 (74.19)
≥5	1399	122 (12.83)	385 (13.42)	185 (18.74)	707 (14.63)
Having elderly members					
Yes	5654	551 (57.94)	1526 (53.21)	686 (69.5)	2891 (59.83)
No	3984	400 (42.06)	1342 (46.79)	301 (30.5)	1941 (40.17)
Having children					
Yes	8304	855 (89.91)	2497 (87.06)	834 (84.5)	4118 (85.22)
No	1334	96 (10.09)	371 (12.94)	153 (15.5)	714 (14.78)
Per capita net household expenditure (US$)					
	9205 ± 8240.78	1097 ± 749.88	2038 ± 1582.33	596 ± 375.19	1414 ± 1233.29
Economic status					
Poorest	1929	93 (9.78)	351 (12.24)	295 (29.89)	1190 (24.63)
Poorer	1930	124 (13.04)	411 (14.33)	265 (26.85)	1130 (23.39)
Middle	1925	172 (18.09)	547 (19.07)	214 (21.68)	992 (20.53)
Richer	1927	243 (25.55)	690 (24.06)	145 (14.69)	849 (17.57)
Richest	1927	319 (33.54)	869 (30.3)	68 (6.89)	671 (13.89)
Age of household head (years)					
	56.34 ± 12.79	57.62 ± 13.96	58.56 ± 13.39	52.29 ± 11.82	55.62 ± 12.08
<45	1850	175 (18.4)	473 (16.49)	270 (27.36)	932 (19.29)
45–60	3883	359 (37.75)	1000 (34.87)	463 (46.91)	2061 (42.65)
≥60	3905	417 (43.85)	1395 (48.64)	254 (25.73)	1839 (38.06)
Gender of household head					
Male	7166	595 (62.57)	1955 (68.17)	800 (81.05)	3816 (78.97)
Female	2472	356 (37.43)	913 (31.83)	187 (18.95)	1016 (21.03)
Education of household head					
Illiteracy	1540	107 (11.25)	326 (11.37)	207 (20.97)	900 (18.63)
Elementary	2801	170 (17.88)	630 (21.97)	348 (35.26)	1653 (34.21)
Middle school	3492	309 (32.49)	1029 (35.88)	351 (35.56)	1803 (37.31)
High school and above	1805	365 (38.38)	883 (30.79)	81 (8.21)	476 (9.85)
Marriage of household head					
Unmarried	257	19 (2)	46 (1.6)	31 (3.14)	161 (3.33)
Married	8125	747 (78.55)	2424 (84.52)	833 (84.4)	4121 (85.29)
Other	1256	185 (19.45)	398 (13.88)	123 (12.46)	550 (11.38)
Working status of household head					
Yes	6634	336 (35.33)	1448 (50.49)	802 (81.26)	4048 (83.77)
No	3004	615 (64.67)	1420 (49.51)	185 (18.74)	784 (16.23)

**Table 2:** Number and proportion of household impoverishment in paying for health care.

Economic Status	2008				2013			
	Average OOP Health Expenditure (US$)	Average Household Total Expenditure (US$)	No. (%) Household with CHE	No. (%) Household with Impoverishment	Average OOP Health Expenditure (US$)	Average Household Total Expenditure (US$)	No. (%) Household with CHE	No. (%) Household with Impoverishment
Poorest	339.50	881.97	195 (50.26)	125 (32.22)	532.89	1359.90	825 (53.54)	589 (38.22)
Poorer	367.41	1383.81	118 (30.33)	47 (12.08)	638.94	2396.46	458 (29.72)	2 (0.13)
Middle	433.35	1946.92	88 (22.8)	0 (0)	710.48	3443.35	271 (17.61)	0 (0)
Richer	458.53	2533.89	47 (12.11)	0 (0)	923.47	4826.28	226 (14.68)	0 (0)
Richest	502.55	3944.31	19 (4.91)	0 (0)	995.11	7991.74	74 (4.81)	0 (0)

Note: OOP, out-of-pocket; CHE, catastrophic health expenditure.

**Table 3:** Results of multilevel logistic regression model.

Variables	Null Model					Random Intercept Model				
	OR	Std. Err.	*p*	95% CI		OR	Std. Err.	*p*	95% CI	
				Lower	Upper				Lower	Upper
Time						0.02	0.19	0.92	−0.39	0.35
Rural						1.72	0.27	<0.001	1.19	2.24
Time × Rural						0.87	0.28	<0.001	−1.43	−0.32
Household scale										
1						Ref.				
2–4						0.28	0.20	0.17	−0.67	0.12
≥5						0.43	0.26	0.10	−0.94	0.08
Having elderly members						0.14	0.10	0.15	−0.33	0.05
Having children						0.23	0.15	0.14	−0.07	0.53
Poorest economic status						3.86	0.16	<0.001	3.55	4.16
Age of household head (years)										
<45						Ref.				
45–60						−0.20	0.18	0.25	−0.55	0.14
≥60						0.07	0.17	0.69	−0.26	0.40
Female Household head						0.05	0.15	0.75	−0.33	0.24
Education of household head										
Illiteracy						Ref.				
Elementary						0.31	0.12	0.01	0.07	0.55
Middle school						0.06	0.14	0.66	−0.22	0.34
High school and above						0.01	0.18	0.97	−0.35	0.37
Marital status of household head										
Unmarried						Ref.				
Married						0.06	0.31	0.86	−0.56	0.67
Others						0.05	0.30	0.87	−0.55	0.64
Household head employed						0.19	0.14	0.17	−0.45	0.08
Diabetes						0.30	0.16	0.06	−0.02	0.62
Hypertension						0.08	0.09	0.39	−0.10	0.25
Intercept						4.85	0.51	<0.001	−5.86	−3.84
Random-effects										
Level 3: county	0.76	0.07		0.64	0.90	0.28	1.25		0.001	1576.38
Level 2: town	0.74	0.05		0.65	0.84	0.50	0.42		0.42	0.59

Note: Null model is a model without any covariate but only an intercept and random effects. From the null model results, we found there was statistical significance in variances at the county and town levels. Ref reference group; OR, odds ratio; CI, confidence interval; Std. Err., Standard error.

**Table 4:** The effect of the New Health Care Reform (NHCR) on impoverishment by residential location.

Comparison	OR	*p*	95% CI	
			Lower Limits	Upper Limits
(Before vs. after reform) in Urban	0.83	0.35	0.56	1.23
(Before vs. after reform) in Rural	0.54	<0.001	0.43	0.68

## List of Abbreviations

NHFPC	National Health and Family Planning Commission of China
NHCR	New Health Care Reform
NHSS	National Health Service Surveys
WB	The Word Bank
WHO	World Health Organization
UEBMI	Urban Employee Basic Medical Insurance
URBMI	Urban Resident Basic Medical Insurance
NRCMI	New Rural Cooperative Medical Insurance
